# Circulating methylated *THBS1* DNAs as a novel marker for predicting peritoneal dissemination in gastric cancer

**DOI:** 10.1002/jcla.23936

**Published:** 2021-08-13

**Authors:** Xuan‐Yu Hu, Zhe‐Nan Ling, Lian‐Lian Hong, Qi‐Ming Yu, Pei Li, Zhi‐Qiang Ling

**Affiliations:** ^1^ Department of Pathophysiology School of Basic Medical Sciences Zhengzhou University Zhengzhou China; ^2^ Experimental Research Centre Institute of Cancer and Basic Medicine (ICBM) Chinese Academy of Sciences Cancer Hospital of the University of Chinese Academy of Sciences Gongshu District, Hangzhou China; ^3^ Department of Hepatobiliary & Pancreatic Surgery The First Affiliated Hospital Zhejiang University School of Medicine Shangcheng District, Hangzhou China

**Keywords:** gastric carcinoma, methylation, peritoneal dissemination, prognosis, *THBS1 gene*

## Abstract

**Objectives:**

Thrombospondin 1 (*THBS1)* is known to play a key role in tumor metastasis, and aberrant DNA methylation is one of the mechanisms regulating *THBS1*. The present study investigated whether methylated *THBS1* in circulating cell‐free DNA from preoperative peritoneal lavage fluid (PPLF) and peripheral blood could be used as a potential biomarker for predicting peritoneal dissemination in gastric cancer (GC) patients.

**Methods:**

The status of *THBS1* methylation was detected by quantitative methylation‐specific PCR (MSP) in tumor tissues, paired PPLF, and serum from 92 GC patients. The correlation between methylated *THBS1* levels and peritoneal dissemination of GC was studied, and its diagnostic value for predicting peritoneal dissemination was clarified by the receiver operating characteristic (ROC) curve.

**Results:**

Aberrant *THBS1* methylation in tumor tissues was significantly higher than that in paracancerous normal tissues (*p* < 0.0001). No *THBS1* methylation was found in 40 healthy controls, and partial methylation was detected in 3 of 48 patients with chronic non‐atrophic gastritis. The frequency of *THBS1* methylation in pairing PPLF and serum from 92 GC patients was 52.2% (48/92) and 58.7% (54/92), respectively. The results of methylated *THBS1* in pairing PPLF and serum were similar to those of tumor tissues. Aberrant *THBS1* methylation in tumor tissues and pairing PPLF or serum was closely related to peritoneal dissemination, tumor progression, and poor prognosis (all *p* < 0.0001).

**Conclusion:**

Circulating methylated *THBS1* DNAs in PPLF/serum may predict peritoneal dissemination, a potential poor prognostic factor for GC patients.

## Graphical abstract

Postoperative recurrence of gastric cancer usually occurs in the peritoneum, and peritoneal dissemination and recurrence are the main causes of poor prognosis. Therefore, it is very necessary to find a biomarker that can predict peritoneal dissemination of gastric cancer cells. Here, we found that THBS1 methylation levels in preoperative serum or peritoneal fluid can accurately predict peritoneal dissemination and suggest a poor prognosis in patients with gastric cancer.

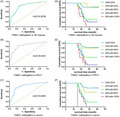

## 1 INTRODUCTION

Gastric cancer (GC) is one of the most common gastrointestinal malignancies, severely threatening human health. According to the latest global cancer burden date of the World Health Organization's International Cancer Research Institute (IARC), there were 4.57 million new cancer cases including 0.48 million GC patients and 3 million cancer deaths including 0.37 million GC patients, which ranks as the third in China.^1^ Metastasis is the main cause of death in patients with advanced GC. More than 40% of GC patients will have metastasis, which seriously affects the treatment, and the 5 years survival rate is less than 30% among GC patients.^1^, ^2^, ^3^

Peritoneal dissemination may exist in early GC, which may not be curative by surgical treatment, leading to poor prognosis.^4^ Peritoneal metastasis is the most common event in recurrent GC.^5^, ^6^ Preoperative peritoneal lavage fluid (PPLF) cytology is usually detected by Papanicolaou staining.^7^ However, peritoneal metastasis sometimes occurs, even when cytological examination shows negative. Therefore, it is necessary to develop novel technologies or to find potential tumor markers for the diagnosis of peritoneal dissemination in GC patients, which is also helpful to improve the treatment of patients with metastatic GC. A series of biomarkers detected by reverse transcription‐polymerase chain reaction (RT‐PCR) in PPLF can improve peritoneal dissemination diagnosis. Common biomarkers include carcinoembryonic antigen (*CEA*),^8^, ^9^ carbohydrate antigen 125 (*CA12*‐*5*),^10^ cytokeratin 20 (*CK20*),^11^ epithelial cell adhesion molecule–specific antigen (clone one: *Ber*‐*EP4*),^12^ matrix metallopeptidase 7(*MMP*‐*7*), ^13^ *Survivin*,^14^ Mucin‐2 (*MUC2*),^15^ interleukin‐17 (*IL*‐*17*),^16^ fatty acid binding protein 1 (*FABP1*),^15^ trefoil factor family 1 (*TFF1*),^15^ and mammary serine protease inhibitor (*MASPIN*).^15^ Most diagnostic biomarkers of peritoneal dissemination are also prognostic markers. Positivity of multiple mRNA markers could predict peritoneal recurrence and poor outcomes in GC patients with cytology negative.

Recently, methylation‐specific PCR (MSP) technology has been successfully used to analyze methylation markers in liquid biopsy specimens, including PPLF samples for peritoneal metastasis diagnosis.^17^, ^18^, ^19^ Our previous studies suggest that DNA methylation in PPLF or serum may be a potential marker for detecting peritoneal dissemination in GC patients.^20^, ^21^, ^22^, ^23^, ^24^, ^25^

Thrombospondin 1 (*THBS1*) is a subunit of the disulfide chain homologous trimer protein and a cohesive glycoprotein that mediates cell‐to‐cell and cell‐to‐matrix interactions. *THBS1* can bind to fibrinogen, fibronectin, laminin, type V collagen, and integrin‐V/‐1, which has been proven to play an important role in angiogenesis, platelet aggregation, and tumorigenesis.^26^ However, the role of *THBS1* in tumors remains somewhat controversial. Some studies reveal that *THBS1* promotes tumor cell invasion and metastasis in breast, gastric, and pancreatic cancers.^27^, ^28^, ^29^ Another study suggests that inhibition of tumor growth by *THBS1* is thought to be associated with its antiangiogenic activity.^30^ Aberrant *THBS1* methylation was detected in gastric cardia adenocarcinoma, melanoma, colorectal cancer, and so on, which is believed to promote tumorigenesis through its effect on angiogenesis.^31^, ^32^, ^33^ In the present study, we explored the methylation status of *THBS1* in GC and its clinicopathological significance, especially the diagnostic value of methylated *THBS1* DNAs in liquid biopsy samples for peritoneal dissemination.

## 2 MATERIALS AND METHODS

### 2.1 Clinical tissue specimens and related information collection

The studies were performed in accordance with the Declaration of Helsinki and approved by the Medical Ethics Committee at Zhejiang Cancer Hospital (Ethical Certification No. zjzlyy [2013]‐03–79, zjzlyy [2015]‐02–125, and zjzlyy‐IRB‐2019–98), and signed written informed consent was provided by patients and their families.

92 histopathologically confirmed primary GC patients were included in this study, which were recruited in Zhejiang Cancer Hospital, Zhejiang Provincial People's Hospital, and Chunan First People's Hospital between January 2008 and December 2009. Details of these patients are summarized below (Table 1). None of these GC cases received any treatment prior to surgery. Collected tumor tissue samples were confirmed by histopathological diagnosis as primary GC, and paired paracancerous histological normal tissues (PCHNTs) must be at least 5 cm from the tumor boundary. PPLF from 92 GC patients was collected in the operating room, as described previously,^21^, ^24^ clarified by centrifugation at 1500 rpm for 10 min at 4°C. DNAs from PPLF specimens were extracted for analysis of *THBS1* gene methylation, and some sediments were coated on one or more glass slides and stained with Papanicolaou (Lafayette). The cytological examination was performed by cytopathologists based on the cell characteristics. In addition, paired blood samples were collected from all recruited GC patients. As a measure of prognosis in cancer patients, we analyzed disease‐free survival (DFS) in patients with GC, defined as the time from surgery to the first postoperative recurrence, or death by GC, or a last contact. The cross‐sectional imaging and serum tumor markers, such as *CEA*, *CA199*, *CA72*‐*4*, and *pepsinogen*, were used to monitor the recurrence and metastasis of GC patients. The follow‐up of all enrolled GC patients was performed periodically until the end date of the project.

**TABLE 1:** Clinicopathological correlations of THBS1 methylation in GC tissues, paired PPLF and serum

Clinicopathological parameters	Number of cases	GC tissues		χ^2^ *p*‐values	PPLF		χ^2^ *p*‐values	Serum		χ^2^ *p*‐values
		U	M		U	M		U	M	
Gender										
Male	53	22	31	0.087	22	31	0.002	24	29	0.324
Female	39	15	24	0.832	16	23	1.000	20	19	0.674
Age at diagnosis										
<60	68	26	42	0.426	26	42	1.013	30	38	1.437
≥60	24	11	13	0.629	12	12	0.343	14	10	0.246
H. *pylori* infection										
Negative	50	22	28	0.652	21	29	0.022	26	24	0.765
Positive	42	15	27	0.523	17	25	1.000	18	24	0.409
Lesion site										
Cardia	30	11	19	0.233	11	19	0.395	15	15	0.084
Body/antrum	62	26	36	0.658	27	35	0.653	29	33	0.826
Tumor size										
<5 cm	60	30	30	6.866	31	29	7.640	34	26	5.403
≥5 cm	32	7	25	0.013	7	25	0.007	10	22	0.028
Growth type										
Swell	20	11	9	2.323	12	8	3.684	13	7	3.021
Infiltration	72	26	46	0.197	26	46	0.073	31	41	0.128
Differentiation										
Well/moderate	62	33	29	13.382	33	29	11.146	39	23	17.321
Poor	30	4	26	0.000	5	25	0.001	5	25	0.000
PPLF cytology										
Negative	53	32	21	21.134	33	20	22.655	37	16	24.218
Positive	39	5	34	0.000	5	34	0.000	7	32	0.000
Lymphatic invasion										
Negative	67	33	34	8.374	33	34	6.427	39	28	10.652
Positive	25	4	21	0.004	5	20	0.016	5	20	0.002
Venous invasion										
Negative	69	34	35	9.419	35	34	10.103	41	28	14.869
Positive	23	3	20	0.003	3	20	0.001	3	20	0.000
T stage										
T1/T2	38	24	14	14.171	26	12	19.636	28	10	17.348
T3/T4	54	13	41	0.000	12	42	0.000	16	38	0.000
Lymph node metastasis										
N0	46	29	17	19.937	30	16	21.700	34	12	25.091
N1‐3	46	8	38	0.000	8	38	0.000	10	36	0.000
Distant metastasis										
M0	85	37	48	3.447	38	47	5.332	44	41	5.026
M1	7	0	7	0.039	0	7	0.039	0	7	0.013
Clinical stage										
Stage I/II	46	30	16	23.915	31	15	25.825	34	12	25.091
Stage III/IV	46	7	39	0.000	7	39	0.000	10	36	0.000

Besides that, antral mucosa biopsy specimens from 88 non‐cancer volunteers were randomly collected by gastroscopy served as controls (54 male and 34 female; mean age 52.9 years). Among these volunteers, 48 patients were diagnosed with chronic, non‐atrophic gastritis, and 40 people were diagnosed with health checkup without other digestive system diseases. Paired blood samples and related information were collected from 88 non‐cancer volunteers. All volunteers provided written informed consent.

### 2.2 PCR diagnosis of Helicobacter pylori (H. *pylori* or Hp) infection

DNAs were extracted from gastric biopsies from 88 volunteers and tumor tissues from 92 GC patients, as described previously, with minor modifications.^34^ DNA was amplified using primers specific for cytotoxin‐associated antigen A (*CagA*), and for H. *pylori* 16S ribosomal RNA (*16S rRNA*). The primer sequence of *CagA* was as follows: forward: 5'‐TTGACCAACAACCACAAACCGAAG‐3'; reverse: 5'‐CTTCCCTTAA TTGCGAGATTCC‐3'.^35^ For H. *pylori 16S rRNA*, the forward sequence was as follows: Hp1‐5'‐CTGGAGAGACTAAGCCCTCC‐3'; Hp2‐5'‐ATTACTGACGCTG ATTGTGC‐3'.^36^ H. *pylori* infection is identified when one of the two tests is positive.

### 2.3 Quantitative MSP analysis

DNAs were extracted from gastric biopsies from 88 volunteers and tumor tissues from 92 GC patients, as described previously,^21^, ^22^, ^23^, ^24^ wherein the collected target cells were treated with 200 μg/ml proteinase K (Sigma‐Aldrich Inc., P O Box 355, Milwaukee, Wisconsin 53201) at 42°C for 72 h. The DNA in serum was extracted using the AxyPrep Blood Genomic DNA Mini Kit (Axygen Biosciences, 33210 Central Avenue, Union City, CA 94587). Bisulfite modification of genomic DNAs was performed by EZ DNA Methylation‐Gold™ Kit (Zymo Research, 625 West Katella Avenue, Suite 30 Orange, CA 92867–4619) following the operating manual's instructions. The specific primers for the methylation and non‐methylation of *THBS1* gene were designed as reported previously.^37^ The primer sequences of *THBS1* methylation were as follows: forward: 5'‐TTGAGTACGTTAAGGTTGCGTGGGC‐3', reverse: 5'‐TA AAAACACTAAAACTACCAATACACCAAA‐3'; the size of the amplification product is 212 bp.^37^ The primer sequences of *THBS1* unmethylation were as follows: forward: 5'‐GGTTGAGTATGTTAAGGTTGTGTGGGT‐3', reverse: 5'‐TAAAAACACTAAAACTACCAATACACCAAA‐3'; the size of amplification products is 230 bp.^37^ Modified DNA samples were analyzed by quantitative MSP on ABI7500 PCR (Applied Biosystems, 850 Lincoln Centre Drive, 94404) by SYBR Premix Taq ExTaq Kit (TaKaRa Bio Inc., Nojihigashi 7–4–38). The proportion of *THBS1* methylation in the samples was calculated according to our previous reports.^20^, ^21^, ^22^, ^23^, ^24^, ^25^ In the present study, the cutoff threshold for *THBS1* methylation was set to 20% based on the control normal samples and the internal quality controls provided in quantitative MSP analysis.

### 2.4 Statistical analysis

Statistical Product and Service Solutions (SPSS) 23.0 statistical software (SPSS Inc.) was adopted for all data analyses. The difference among groups was analyzed using the paired *t* test for normal distribution by Fisher's exact test. The Kaplan‐Meier method was adopted for survival analysis in patients with GC, and the log‐rank test was used for assessing significance. The identified prognostic factors were determined by univariate analysis with the Cox regression model (proportional hazards model), and the combined effects were explored by multivariate analysis with the Cox regression model. *p*‐value <0.05 was considered statistically significant.

## 3 RESULTS

### 3.1 Frequency of *THBS1* methylation in GC tissues, paired blood, and PPLF samples

The incidence of *THBS1* promoter methylation was first analyzed in GC tissues and paired adjacent normal tissues and was estimated as 59.8% (55/92) and 15.2% (14/92) for paired PCHNT tissues (*p* < 0.0001). Also, the levels of *THBS1* methylation in the non‐cancer control group were examined. The results showed that *THBS1* methylation was detected in 6.3% (3/48) patients with chronic non‐atrophic gastritis, while no methylation was detected in 40 healthy individuals. The incidence of *THBS1* methylation was significantly higher in GC tissues than that in the non‐cancer control group (*p* < 0.0001), with no significant difference found in the incidence of *THBS1* methylation between PCHNTs and controls (*p*>0.05). These results suggested that *THBS1* methylation may be a potential marker for GC patients.

**FIGURE 1:**
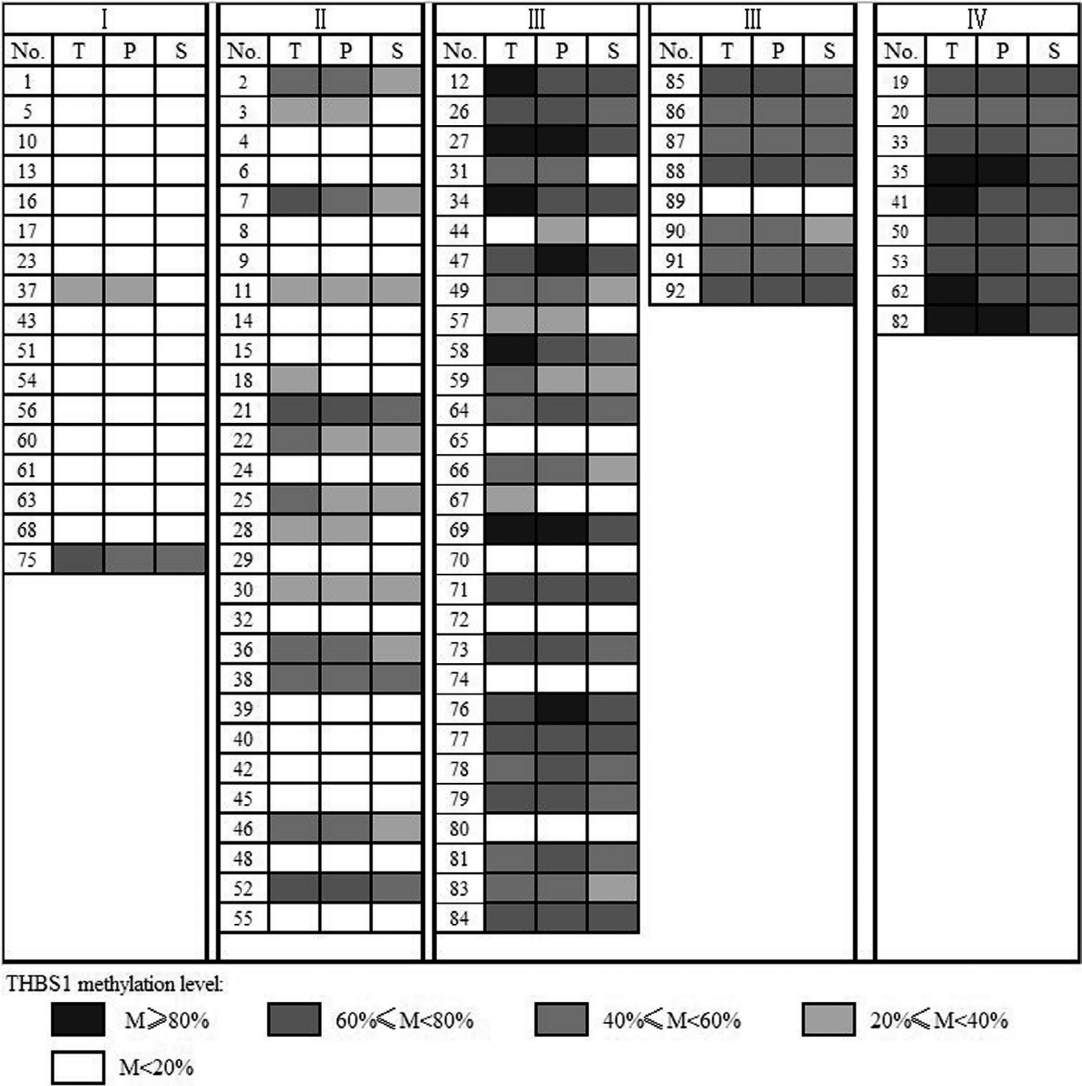
Comparison of *THBS1* methylation in GC tissues, paired preoperative peritoneal lavage fluid (PPLF), and serum samples (T: tumor tissues; P: preoperative peritoneal lavage fluid (PPLF); and S: serum). The figure only showed % methylation of tumor tissue in GC, and Ⅰ‐Ⅳ represent the clinical staging

The incidence of *THBS1* methylation was subsequently examined in paired PPLF and serum samples. The results showed that 98.2% (54/55) GC patients with *THBS1* methylation exhibited the same alteration in their PPLF DNAs, and 87.3% (48/54) GC patients with *THBS1* methylation demonstrated the identical alteration in corresponding serum DNAs, while no *THBS1* methylation was detected in corresponding PPLF and serum specimens in 37 GC patients with negative *THBS1* methylation in tumor tissues (Figure 1).

**FIGURE 2:**
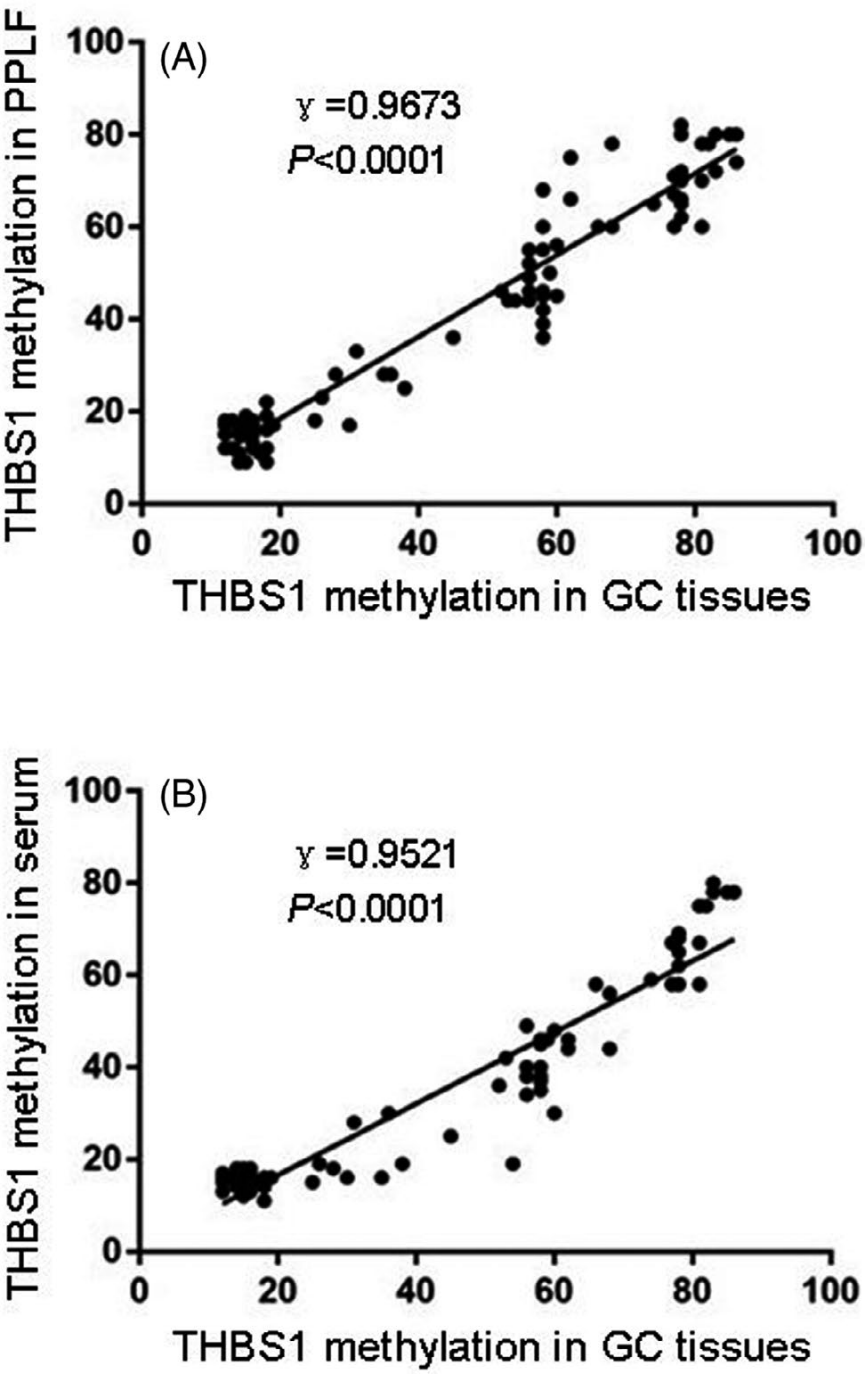
Correlation analysis of *THBS1* methylation results between GC tissues and paired PPLF or paired serum (A: correlation of *THBS1* methylation results between GC tissues and paired PPLF (γ = 0.9673, *p* < 0.0001); B: correlation of *THBS1* methylation results between GC tissues and paired serum (γ = 0.9521, *p* < 0.0001))

Among 54 GC patients with positive *THBS1* methylation in PPLF samples, 72.2% (39/54) patients showed positive PPLF cytology, and 97.1% (33/34) patients with both positive cytology and *THBS1* methylation in tumor tissues showed *THBS1* methylation in paired PPLF samples. The correlation analysis revealed that *THBS1* methylation in PPLF was positively correlated with *THBS1* methylation in tumor tissue (γ = 0.9673, *p* < 0.0001) and *THBS1* methylation in serum was also positively correlated with methylation in the tumor tissue (γ = 0.9521, *p* < 0.0001) (Figure 2).

**FIGURE 3:**
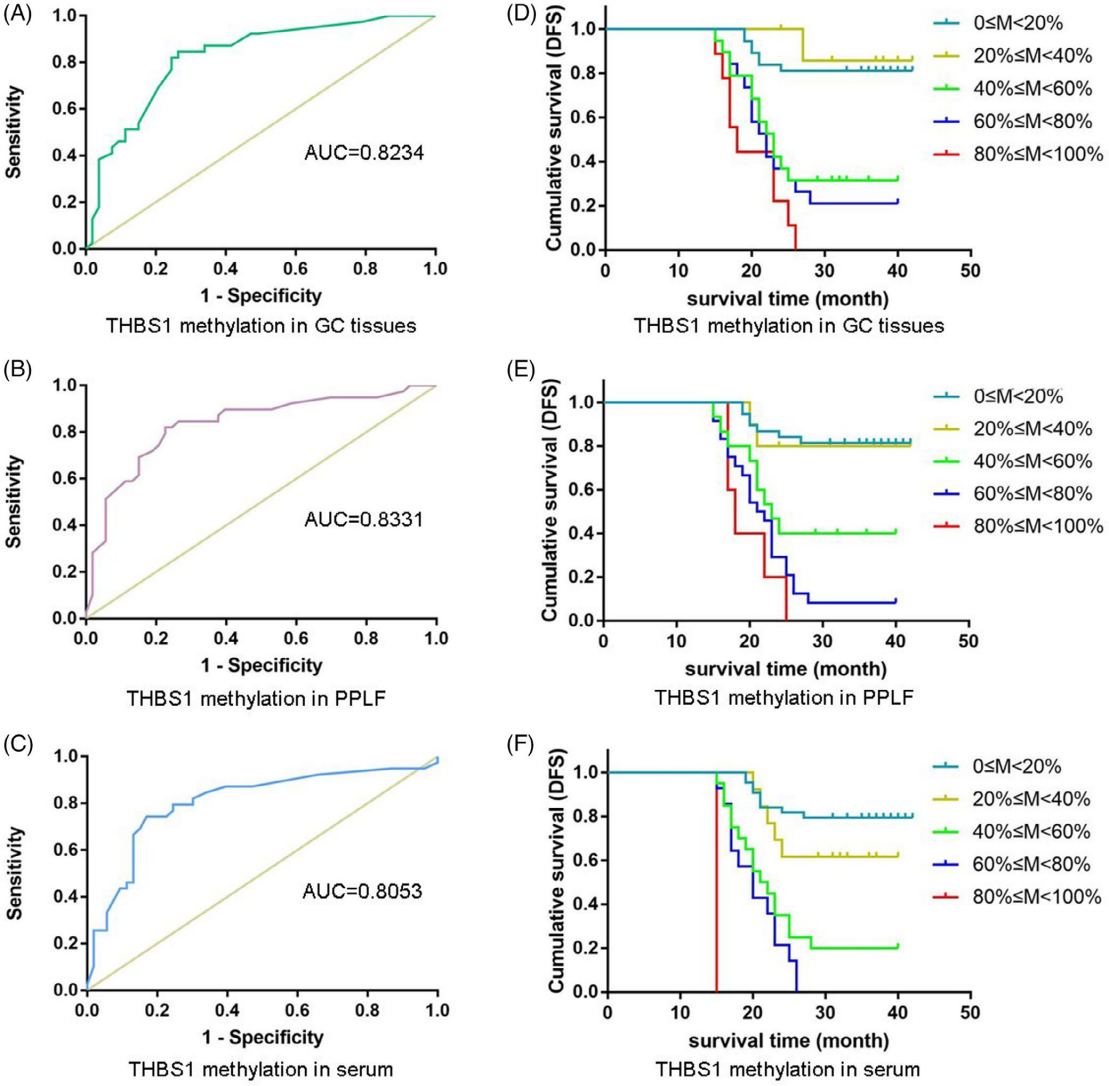
Diagnostic value of *THBS1* methylation in GC tissues, PPLF, and serum for the peritoneal dissemination of GC and its effect on patient prognosis (A–C): The diagnostic value of *THBS1* methylation in GC tissues, paired PPLF, and serum for peritoneal dissemination of GC was determined by ROC curves, of which the Aζ value of the ROC curve was 0.8234 for GC tissues, 0.8331 for PPLF, and 0.8053 for serum compared with positive PPLF cytology. (D–F): *THBS1* methylation in GC tissues, PPLF, and serum correlated with patient prognosis. Cumulative DFS curves were plotted against *THBS1* methylation level in GC tissues (D), PPLF (E), and serum (F). In D, E, and F, Kaplan‐Meier analysis was used, and all parameters were *p* < 0.0001

The accuracy of methylated *THBS1* DNAs in PPLF samples for the diagnosis of GC peritoneal dissemination was 63.0% (34/54), with a sensitivity of 87.2% (34/39) and a specificity of 100% (34/34). Moreover, the accuracy of methylated *THBS1* DNAs in the serum samples for the diagnosis of GC peritoneal dissemination was 66.7% (32/48), with a sensitivity of 82.1% (32/39) and a specificity of 100% (32/32). Strikingly, positive PPLF cytology was served as a gold standard, and the diagnostic value of *THBS1* methylation in GC tissues, paired PPLF, and serum for the peritoneal dissemination of GC was determined based on the ROC curves, of which the Aζ value of the ROC curve was 0.8234 for tumor tissues (Figure 3A), 0.8331 for PPLF (Figure 3B), and 0.8053 for serum (Figure 3C) compared with positive PPLF cytology. The results showed that *THBS1* methylation in PPLF and serum is of high diagnostic value for peritoneal dissemination, suggesting that *THBS1* methylation in liquid biopsy samples served as a novel marker for peritoneal dissemination in patients with GC.

### 3.2 Level of *THBS1* methylation is closely related to progression in GC patients

The clinicopathological correlation analysis of *THBS1* methylation in GC tissues, paired PPLF, and serum was summarized below (Table 1). Results showed that *THBS1* methylation levels in GC tissues, paired PPLF, and serum were significantly associated with tumor size, histological differentiation, lymphatic invasion, venous invasion, invasive depth, lymph node involvement, distant metastasis, and clinical staging, respectively (all *p* < 0.05). Specifically, high levels of *THBS1* methylation in PPLF and serum were closely associated with the positive results predicted by peritoneal lavage cytology (PLC), suggesting that *THBS1* methylation in liquid biopsy samples could serve as a valuable marker for peritoneal dissemination in GC. However, abnormal *THBS1* methylation in tumor tissue or PPLF/serum was not associated with gender, age of diagnosis, *H*. *pylori* infection, and lesion site and growth type (all *p*>0.05).

Among 92 GC patients, 39 (42.4%) showed positive PPLF cytology, which was significantly related to the TNM staging (Table 1). Of 54 GC patients with stage T3/T4, 35 showed positive PPLF cytology, with a positive rate of 64.8%. The positive rate of cytology in stage T1/T2 was only 10.5% (4/38), which was significantly different from that in T3/T4 stage (*p* < 0.0001). Furthermore, the PPLF cytology positive rate was 82.6% (38/46), significantly higher than that in those with lymph node negative, whose PPLF cytology positive rate was 2.2% (1/46), indicating a significant difference between the two groups (*p* < 0.0001). The positive rate of PPLF cytology was 100% in GC patients with the M1 stage. These results suggested that the positive rate of PPLF cytology increases proportionally when the tumor invades the deep layers of the gastric wall or lymph nodes and when the tumor loses differentiation.

### 3.3 Circulating methylated *THBS1* DNAs is a novel adverse prognostic factor in GC patients

Univariate analysis showed that *THBS1* methylation levels in tumor tissues or PPLF or serum were significantly associated with adverse prognosis in GC (all *p* < 0.0001). Moreover, tumor size (*p* = 0.003), growth type (*p* = 0.004), histological differentiation (*p* < 0.0001), venous invasion (*p* = 0.001), *THBS1* methylation in tumor tissues (*p* < 0.0001), or PPLF (*p* < 0.0001), or serum (*p* < 0.0001), and PLC positive (*p* < 0.0001) were significantly correlated with DFS of GC patients (Table 2). However, no significant correlation was established between the DFS of GC patients and gender (*p* = 0.625), age (*p* = 0.228), *H*. *pylori* infection (*p* = 0.414), and tumor site (*p* = 0.783) (Table 2).

**TABLE 2:** Univariate survival analysis of clinicopathologic data of 92 GC cases

Variables	Hazard ratio	95% confidence interval	*p*‐value
Gender			
(Female/Male)	1.162	0.636–2.125	0.625
Age			
(<60/≥60)	0.638	0.307–1.325	0.228
H. *pylori* infection			
(Negative/Positive)	1.276	0.711–2.289	0.414
Tumor size			
(<5 cm/≥5 cm)	2.425	1.350–4.359	0.003
Growth type			
(swell/infiltration)	18.241	2.509–132.612	0.004
Histological differentiation			
(well and moderate/poor)	3.470	1.915–6.288	0.000
Lesion site			
(body and antrum/cardia)	0.918	0.498–1.690	0.783
Venous invasion			
(Negative/Positive)	2.834	1.546–5.193	0.001
PLC			
(Negative/Positive)	27.139	10.822–68.058	0.000
THBS1 in tumor tissues			
(UM/M)	7.191	3.326–15.551	0.000
THBS1 methylation in PPLF			
(UM/M)	7.283	3.478–15.250	0.000
THBS1 in serum			
(UM/M)	6.627	3.186–12.599	0.000

Abbreviations: PLC, Peritoneal lavage cytology; PPLF, Preoperative peritoneal lavage fluid; M, Methylation; UM, Unmethylation.

The average time of DFS was 25.1 months in 55 GC patients with *THBS1* methylation and 35.1 months in 37 GC patients with *THBS1* unmethylation in tumor tissues, indicating a significant difference (*p* < 0.01). *THBS1* methylation levels in GC tissues, PPLF, or serum were significantly associated with adverse prognosis in GC and worsened with the increase in *THBS1* methylation degree (Figure 3D–F).

Multivariate survival analysis revealed an independent survival disadvantage in GC patients with *THBS1* methylation in tumor tissue, PPLF, or serum specimens (*p* < 0.01) (Table 3). In addition, PLC‐positive (*p* = 0.000) or growth type (*p* = 0.009) parameter could be an independent influencing factor of prognosis in GC patients after surgery (Table 3). However, no significant association was established between DFS of GC patients and gender (*p* = 0.664) or age (*p* = 0.381) or *H*. *pylori* infection (*p* = 0.920) or tumor site (*p* = 0.873) or tumor size (*p* = 0.063) or histological differentiation (*p* = 0.733) or venous invasion (*p* = 0.089) (Table 3).

**TABLE 3:** Multivariate survival analysis of clinicopathologic data of 92 GC cases

Variables	Hazard ratio	95% confidence interval	*p*‐value
Gender			
(Female/Male)	1.152	0.608–2.181	0.664
AGE			
(<60/≥60)	0.705	0.323–1.540	0.381
H. *pylori* infection			
(Negative/Positive)	0.968	0.513–1.827	0.920
Tumor size			
(<5 cm/≥5 cm)	1.837	0.967–3.488	0.063
Growth type			
(Swell/Infiltration)	14.639	1.945–110.147	0.009
Histological differentiation			
(Well and moderate/Poor)	1.132	0.556–2.304	0.733
Lesion site			
(Body and antrum/Cardia)	1.055	0.547–2.036	0.873
Venous invasion			
(Negative/Positive)	1.852	0.910–3.768	0.089
PLC			
(Negative/Positive)	47.165	13.436–165.568	0.000
THBS1 in tumor tissues			
(UM/M)	4.377	1.880–10.190	0.001
THBS1 methylation in PPLF			
(UM/M)	4.015	1.923–8.381	0.000
THBS1 in serum			
(UM/M)	3.838	1.691–8.710	0.001

Abbreviations: PLC, Peritoneal lavage cytology; PPLF, Preoperative peritoneal lavage fluid; M, Methylation; UM, Unmethylation.

## 4 DISCUSSION

The prognosis of patients with advanced GC is poor even after radical surgery.^1^, ^2^, ^3^ Peritoneal dissemination is the most common type of spread, mainly caused by the seeding of free cancer cells from the primary GC. The peritoneum is also the most frequent site of recurrence in GC patients who underwent curative resection.^5^, ^6^ The examination of free tumor cells in PPLF in GC patients revealed peritoneal dissemination, predicted peritoneal recurrence and prognosis, and selected proper treatments.^7^, ^38^ Currently, cytology and molecular biology are the two most popular methods for the detection of peritoneal dissemination.^7^, ^8^, ^9^, ^10^, ^11^, ^12^, ^13^, ^14^, ^15^, ^16^, ^17^, ^18^, ^19^, ^20^, ^21^, ^22^, ^23^, ^24^, ^25^

In previous studies, aberrant DNA promoter methylation of tumor‐related genes has been identified in the body fluids for peritoneal dissemination in GC patients.^20^, ^21^, ^22^, ^23^, ^24^ Promoter methylation of *THBS1* gene has been studied in various malignancies, such as colorectal cancer, melanoma, and GC tissues.^31^, ^32^, ^33^ Quantitative MSP technology has high sensitivity and specificity and can be used to detect the methylation levels of tumor‐related genes in liquid biopsy samples of tumor patients for tumor screening, early diagnosis, metastasis warning, recurrence monitoring, and prognostic assessment.^18^, ^39^ In this study, we did not find any abnormal methylation of the *THBS1* gene in the serum or PPLF DNAs in tumor tissues. Moreover, quantitative MSP can detect *THBS1* methylation in small amounts of tumor DNAs in serum samples.

The RT‐PCR techniques have been used to detect tumor markers in PPLF or blood samples of GC patients to evaluate peritoneal dissemination. Previous studies focused on detecting several tumor‐specific genes, such as *CEA*, *CA12*‐*5*, *CK20*, *Ber*‐*EP4*, *MMP*‐*7*, *Survivin*, *MUC2*, *IL*‐*17*, *FABP1*, *TFF1*, and *MASPIN*.^8^, ^9^, ^10^, ^11^, ^12^, ^13^, ^14^, ^15^ Quantitative MSP method based on the detection of DNA methylation seems to have advantages over RT‐PCR detection of gene transcription levels. The DNA extracted from tumor tissues, PPLF, or serum samples is stable and amplified by PCR techniques. Abnormal DNA methylation represents a chemically and biologically stable cancer‐specific biomarker that is readily detected and independent of levels of gene expression.^40^ Moreover, quantitative MSP method for the detection of DNA methylation is only suitable for tumor tissue, PPLF, or serum specimens with DNA changes, which also demonstrates the specificity of DNA methylation detection.^40^ On the other hand, due to the easy degradation of the mRNA, various factors, such as the sample preservation and the careless operation of the researchers, will affect the detection results of the mRNA.

In this study, the detection of *THBS1* methylation in GC was feasible, with highly consistent levels of *THBS1* methylation detected in serum, PPLF, and tumor tissues in the same patient. In addition, the status of *THBS1* methylation has a high correlation with clinicopathological parameters; that is, it can predict peritoneal dissemination and is more sensitive than peritoneal cytology. Some studies have shown that *THBS1* methylation is an early and frequent event in various tumors that can occur at each stage in the tumor and gradually increases as tumor progresses.^31^, ^32^, ^33^ Our results suggested that *THBS1* methylation is a new marker for the diagnosis of peritoneal transmission and can serve as a novel prognostic biomarker for GC patients. The current results confirmed that circulating methylated *THBS1* DNAs is a potential poor prognostic factor in GC patients.

In conclusion, this was the first prospective study of the epigenetic changes of the *THBS1* gene predicting the peritoneal dissemination in GC patients. Intriguingly, *THBS1* methylation was detected in DNAs extracted from preoperative serum samples in GC patients, which was highly consistent with *THBS1* methylation results detected in PPLF and tumor tissues in the same patient. Also, the detection of *THBS1* promoter methylation is sensitive to the peritoneal dissemination compared with peritoneal cytology. Abnormal *THBS1* methylation was readily detected in preoperative serum samples in GC patients with peritoneal dissemination, and it is therefore considered a valuable biomarker for assessing the risk of peritoneal dissemination and the early diagnosis of peritoneal micrometastasis. All in all, it is potentially significant for evaluating the high risk of peritoneal dissemination and monitoring the clinical treatment and prognosis of GC patients.

## CONFLICTS OF INTEREST

This study is not related to any potentially competing financial or other interests.

## AUTHOR CONTRIBUTIONS

Hu XY, Ling ZN, Hong LL, Li P, and Ling ZQ contributed to the design, execution, and analysis of data. Yu QM provided clinical samples including GC tissue, serum, and PPLF samples. Hu XY, Li P, and Ling ZQ drafted the manuscript. Ling ZN and Hong LL provided some help for data analysis. All the authors were involved in the critical revision of the manuscript.

## Data Availability

The data used to support the findings of this study are available from the corresponding author upon request.

## References

[1] Sung H, Ferlay J, Siegel RL, et al. Global cancer statistics 2020: globocan estimates of incidence and mortality worldwide for 36 cancers in 185 countries. CA Cancer J Clin. 2021;71(3):209–249. 10.3322/caac.21660 33538338

[2] Bray F, Ferlay J, Soerjomataram I, Siegel RL, Torre LA, Jemal A. Global cancer statistics 2018: GLOBOCAN estimates of incidence and mortality worldwide for 36 cancers in 185 countries. CA Cancer J Clin. 2018;68(6):394‐424. 30207593 10.3322/caac.21492

[3] Chen W, Zheng R, Baade PD, et al. Cancer statistics in China, 2015. CA Cancer J Clin. 2016;66(2):115‐132. 26808342 10.3322/caac.21338

[4] Wanebo HJ, Kennedy BJ, Chmiel J, Steele G, Winchester D, Osteen R. Cancer of the stomach. a patient care study by the American college of surgeons. Ann Surg. 1993;218(5):583‐592. 8239772 10.1097/00000658-199321850-00002 PMC1243028

[5] Yoo CH, Noh SH, Shin DW, Choi SH, Min JS. Recurrence following curative resection for gastric carcinoma. Br J Surg. 2000;87(2):236‐242. 10671934 10.1046/j.1365-2168.2000.01360.x

[6] Fujiwara Y, Doki Y, Taniguchi H, et al. Genetic detection of free cancer cells in the peritoneal cavity of the patient with gastric cancer: present status and future perspectives. Gastric Cancer. 2007;10(4):197‐204. 18095074 10.1007/s10120-007-0436-5

[7] Virgilio E, Giarnieri E, Giovagnoli MR, et al. Gastric cancer cells in peritoneal lavage fluid: a systematic review comparing cytological with molecular detection for diagnosis of peritoneal metastases and prediction of peritoneal recurrences. Anticancer Res. 2018;38(3):1255‐1262. 29491048 10.21873/anticanres.12347

[8] Kanetaka K, Ito S, Susumu S, et al. Clinical significance of carcinoembryonic antigen in peritoneal lavage from patients with gastric cancer. Surgery. 2013;154(3):563‐572. 23806263 10.1016/j.surg.2013.03.005

[9] Nakabayashi K, Uraoka T, Shibuya M, Matsuura N, Tsujimoto M. Rapid detection of CEA mRNA in peritoneal washes using one‐step nucleic acid amplification (OSNA®) for gastric cancer patients. Clin Chim Acta. 2015;439:137‐142. 25454718 10.1016/j.cca.2014.10.014

[10] Huang C, Liu Z, Xiao LI, et al. Clinical significance of serum CA125, CA19‐9, CA72‐4, and fibrinogen‐to‐lymphocyte ratio in gastric cancer with peritoneal dissemination. Front Oncol. 2019;9:e1159. 10.3389/fonc.2019.01159 PMC6848261 31750248

[11] Kodera Y, Nakanishi H, Ito S, et al. Prognostic significance of intraperitoneal cancer cells in gastric carcinoma: detection of cytokeratin 20 mRNA in peritoneal washes, in addition to detection of carcinoembryonic antigen. Gastric Cancer. 2005;8(3):142‐148. 16086116 10.1007/s10120-005-0318-7

[12] Lorenzen S, Panzram B, Rosenberg R, et al. Prognostic significance of free peritoneal tumor cells in the peritoneal cavity before and after neoadjuvant chemotherapy in patients with gastric carcinoma undergoing potentially curative resection. Ann Surg Oncol. 2010;17(10):2733‐2739. 20490698 10.1245/s10434-010-1090-4

[13] Yonemura Y, Fujimura T, Ninomiya I, et al. Prediction of peritoneal micrometastasis by peritoneal lavaged cytology and reverse transcriptase‐ polymerase chain reaction for matrix metalloproteinase‐7 mRNA. Clin Cancer Res. 2001;7(6):1647‐1653. 11410502

[14] Dalal KM, Woo Y, Kelly K, et al. Detection of micrometastases in peritoneal washings of gastric cancer patients by the reverse transcriptase polymerase chain reaction. Gastric Cancer. 2008;11(4):206‐213. 19132482 10.1007/s10120-008-0483-6

[15] Mori K, Suzuki T, Uozaki H, et al. Detection of minimal gastric cancer cells in peritoneal washings by focused microarray analysis with multiple markers: clinical implications. Ann Surg Oncol. 2007;14(5):1694‐1702. 17294072 10.1245/s10434-006-9321-4

[16] Iida T, Iwahashi M, Katsuda M, et al. Prognostic significance of IL‐17 mRNA expression in peritoneal lavage in gastric cancer patients who underwent curative resection. Oncol Rep. 2014;31(2):605‐612. 24337702 10.3892/or.2013.2911

[17] Zavridou M, Mastoraki S, Strati A, Tzanikou E, Chimonidou M, Lianidou E. Evaluation of preanalytical conditions and implementation of quality control steps for reliable gene expression and DNA methylation analyses in liquid biopsies. Clin Chem. 2018;64(10):1522‐1533. 30018056 10.1373/clinchem.2018.292318

[18] Zavridou M, Strati A, Bournakis E, Smilkou S, Tserpeli V, Lianidou E. Prognostic significance of gene expression and DNA methylation markers in circulating tumor cells and paired plasma derived exosomes in metastatic castration resistant prostate cancer. Cancers (Basel). 2021;13(4):780. 33668490 10.3390/cancers13040780 PMC7918693

[19] Mastoraki S, Strati A, Tzanikou E, et al. ESR1 Methylation: a liquid biopsy‐based epigenetic assay for the follow‐up of patients with metastatic breast cancer receiving endocrine treatment. Clin Cancer Res. 2018;24(6):1500‐1510. 29284708 10.1158/1078-0432.CCR-17-1181

[20] Yu J‐L, Lv P, Han J, et al. Methylated TIMP‐3 DNA in body fluids is an independent prognostic factor for gastric cancer. Arch Pathol Lab Med. 2014;138(11):1466‐1473. 25357107 10.5858/arpa.2013-0285-OA

[21] Han J, Lv P, Yu J‐L, et al. Circulating methylated MINT2 promoter DNA is a potential poor prognostic factor in gastric cancer. Dig Dis Sci. 2014;59(6):1160‐1168. 24385013 10.1007/s10620-013-3007-0

[22] Ling ZQ, Lv P, Lu XX, et al. Circulating methylated XAF1 DNA indicates poor prognosis for gastric cancer. PLoS One. 2013;8(6):e67195. 23826230 10.1371/journal.pone.0067195 PMC3695092

[23] Lu XX, Yu JL, Ying LS, et al. Stepwise cumulation of RUNX3 methylation mediated by Helicobacter pylori infection contributes to gastric carcinoma progression. Cancer. 2012;118(22):5507‐5517. 22576578 10.1002/cncr.27604

[24] Yu QM, Wang XB, Luo J, et al. CDH1 methylation in preoperative peritoneal washes is an independent prognostic factor for gastric cancer. J Surg Oncol. 2012;106(6):765‐771. 22514028 10.1002/jso.23116 PMC3495294

[25] Ling ZQ, Zhao Q, Zhou SL, Mao WM. MSH2 promoter hypermethylation in circulating tumor DNA is a valuable predictor of disease‐free survival for patients with esophageal squamous cell carcinoma. Eur J Surg Oncol. 2012;38(4):326‐332. 22265839 10.1016/j.ejso.2012.01.008

[26] Silverstein RL, Febbraio M. CD36‐TSP‐HRGP interactions in the regulation of angiogenesis. Curr Pharm Des. 2007;13(35):3559‐3567. 18220792 10.2174/138161207782794185

[27] Yee KO, Connolly CM, Duquette M, Kazerounian S, Washington R, Lawler J. The effect of thrombospondin‐1 on breast cancer metastasis. Breast Cancer Res Treat. 2009;114(1):85‐96. 18409060 10.1007/s10549-008-9992-6 PMC2631620

[28] Lin XD, Chen SQ, Qi YL, Zhu JW, Tang Y, Lin JY. Overexpression of thrombospondin‐1 in stromal myofibroblasts is associated with tumor growth and nodal metastasis in gastric carcinoma. J Surg Oncol. 2012;106(1):94‐100. 22231149 10.1002/jso.23037

[29] Kasper HU, Ebert M, Malfertheiner P, Roessner A, Kirkpatrick CJ, Wolf HK. Expression of thrombospondin‐1 in pancreatic carcinoma: correlation with microvessel density. Virchows Arch. 2001;438(2):116‐120. 11253112 10.1007/s004280000302

[30] Kazerounian S, Yee KO, Lawler J. Thrombospondins in cancer. Cell Mol Life Sci. 2008;65(5):700‐712. 18193162 10.1007/s00018-007-7486-z PMC2752021

[31] Rojas A, Meherem S, Kim YH, et al. The aberrant methylation of TSP1 suppresses TGF‐beta1 activation in colorectal cancer. Int J Cancer. 2008;123(1):14‐21. 18425817 10.1002/ijc.23608 PMC2777657

[32] Lindner DJ, Wu Y, Haney R, et al. Thrombospondin‐1 expression in melanoma is blocked by methylation and targeted reversal by 5‐Aza‐deoxycytidine suppresses angiogenesis. Matrix Biol. 2013;32(2):123‐132. 23202046 10.1016/j.matbio.2012.11.010 PMC3615071

[33] Guo W, Dong Z, He M, et al. Aberrant methylation of thrombospondin‐1 and its association with reduced expression in gastric cardia adenocarcinoma. J Biomed Biotechnol. 2010;2010:721485. 20300551 10.1155/2010/721485 PMC2838370

[34] Sicinschi LA, Correa P, Peek RM, et al. Helicobacter pylori genotyping and sequencing using paraffin‐embedded biopsies from residents of Colombian areas with contrasting gastric cancer risks. Helicobacter. 2008;13(2):135‐145. 18321303 10.1111/j.1523-5378.2008.00554.x PMC2977907

[35] van Doorn LJ, Figueiredo C, Rossau R, et al. Typing of Helicobacter pylori vacA gene and detection of cagA gene by PCR and reverse hybridization. J Clin Microbiol. 1998;36(5):1271‐1276. 9574690 10.1128/jcm.36.5.1271-1276.1998 PMC104813

[36] Ho SA, Hoyle JA, Lewis FA, et al. Direct polymerase chain reaction test for detection of Helicobacter pylori in humans and animals. J Clin Microbiol. 1991;29(11):2543‐2549. 1723072 10.1128/jcm.29.11.2543-2549.1991 PMC270370

[37] Huang C, Zhou X, Li Z, et al. Downregulation of thrombospondin‐1 by DNA hypermethylation is associated with tumor progression in laryngeal squamous cell carcinoma. Mol Med Rep. 2016;14(3):2489‐2496. 27485791 10.3892/mmr.2016.5580 PMC4991671

[38] Chen Y, Zhou Q, Wang H, et al. Predicting peritoneal dissemination of gastric cancer in the era of precision medicine: molecular characterization and biomarkers. Cancers (Basel). 2020;12(8):2236. 10.3390/cancers12082236 PMC7547377 32785164

[39] Lo YMD, Han DSC, Jiang P, et al. Epigenetics, fragmentomics, and topology of cell‐free DNA in liquid biopsies. Science. 2021;372(6538):eaaw3616. 33833097 10.1126/science.aaw3616

[40] Kanyama Y, Hibi K, Nakayama H, et al. Detection of p16 promoter hypermethylation in serum of gastric cancer patients. Cancer Sci. 2003;94(5):418‐420. 12824886 10.1111/j.1349-7006.2003.tb01457.x PMC11160308

